# Sirt6 cooperates with Blimp1 to positively regulate osteoclast differentiation

**DOI:** 10.1038/srep26186

**Published:** 2016-05-18

**Authors:** So Jeong Park, Jeong-Eun Huh, Jihye Shin, Doo Ri Park, Ryeojin Ko, Gyu-Rin Jin, Dong-Hyun Seo, Han-Sung Kim, Hong-In Shin, Goo Taeg Oh, Hyun Seok Kim, Soo Young Lee

**Affiliations:** 1 Department of Life Science, Ewha Womans University, Seoul 120-750, Korea; 2 The Research Center for Cellular Homeostasis, Ewha Womans University, Seoul 120-750, Korea; 3 Department of Biomedical Engineering, College of Health Science, Institute of Medical Engineering, Yonsei University, Wonju, Korea; 4 IHBR, Department of Oral Pathology, School of Dentistry, Kyungpook National University, Daegu 700-412, Korea; 5 Department of Bioinspired Science, Ewha Womans University, Seoul 120-750, Korea

## Abstract

Global deletion of the gene encoding a nuclear histone deacetylase sirtuin 6 (Sirt6) in mice leads to osteopenia with a low bone turnover due to impaired bone formation. But whether Sirt6 regulates osteoclast differentiation is less clear. Here we show that Sirt6 functions as a transcriptional regulator to directly repress anti-osteoclastogenic gene expression. Targeted ablation of Sirt6 in hematopoietic cells including osteoclast precursors resulted in increased bone volume caused by a decreased number of osteoclasts. Overexpression of Sirt6 led to an increase in osteoclast formation, and *Sirt6*-deficient osteoclast precursor cells did not undergo osteoclast differentiation efficiently. Moreover, we showed that Sirt6, induced by RANKL-dependent NFATc1 expression, forms a complex with B lymphocyte-induced maturation protein-1 (Blimp1) to negatively regulate expression of anti-osteoclastogenic gene such as *Mafb*. These findings identify Sirt6 as a novel regulator of osteoclastogenesis by acting as a transcriptional repressor.

Osteoclasts are multinucleated myeloid lineage cells that degrade bone matrix^1^. The maintenance of bone homeostasis depends on a delicate balance between bone-resorbing osteoclasts and bone-forming osteoblasts^2^,^3^. Excessive bone resorption by osteoclasts is often associated with bone and joint diseases, such as osteoporosis and rheumatoid arthritis^4^,^5^,^6^,^7^. Therefore, as proper bone homeostasis requires tight regulation of osteoclast differentiation, studies on the molecular mechanisms of osteoclast differentiation are important in the understating the pathophysiology of the skeletal system.

Activation of transcription factors such as microphthalmia transcription factor (MITF), c-Fos, nuclear factor-κB (NF-κB), and nuclear factor of activated T-cells, cytoplasmic1 (NFATc1) is required for optimal osteoclast differentiation. In particular, NFATc1 is known to the essential factor of osteoclastogenesis and is induced by receptor activator of NF-κB ligand (RANKL) and immunoreceptor tyrosine-based activation motif (ITAM) signals^2^,^3^. NFATc1 works together with other transcription factors, such as AP1, PU.1, MITF, and CREB to induce various osteoclast-specific genes, including *Dc-stamp*^8^ and *Atp6v0d2*^9^ in addition to a number of genes such as *Acp5, Calcr*, and *Itgb3*^10^.

Recent reports indicate that osteoclastogenesis is repressed by transcriptional repressors which are expressed and functional in osteoclast precursors^10^. These include MafB, IRF8, and Bcl6 that inhibit osteoclast differentiation mainly through the suppression of NFATc1 expression and activity^11^. Thus, the expression of such transcriptional repressors needs to be repressed for osteoclast differentiation to proceed efficiently. More recently, it has been reported that these transcriptional repressors are coordinately downregulated by other transcriptional repressor Blimp1, which is induced by the RANKL-NFATc1 axis during osteoclastogenesis^12^.

Sirtuins have been linked to metabolic regulation, stress tolerance, and aging^13^,^14^,^15^. Mammals have seven Sirtuins (Sirt1-Sirt7), found in different subcellular compartments, including the nucleus (Sirt6 and Sirt7), and mitochondria (Sirt3, Sirt4 and Sirt5). Sirt1 and Sirt2 are found both in the nucleus and the cytoplasm and in a cell and tissue-dependent manner^16^,^17^. Of interest, Sirt6 is known to be a chromatin-associated nuclear protein regulating genomic stability, cellular metabolism, inflammation, stress response and longevity^18^,^19^,^20^,^21^,^22^,^23^. *Sirt6*-deficient (*Sirt6*^−/−^) mice suffer from a variety degenerative aging phenotypes and die around 4 weeks after birth^18^,^19^. In addition, *Sirt6*^−/−^ mice exhibit osteopenia due to impaired mainly bone formation, with 30% reduction in bone mineral density. Since bones are still developing in mice at this age, early postnatal lethality of *Sirt6*^−/−^ mice precludes investigation of Sirt6 function in adult mice^19^ and makes it difficult to distinguish developmental versus bone remodeling defects in bone metabolism.

Here we investigated the function of Sirt6 in osteoclastogenesis by disrupting *Sirt6* at an adult stage using *Mx1-Cre* mice. We found that Sirt6 induced by RANKL-NFATc1 axis acted as a transcriptional repressor of negative regulators of NFATc1 during osteoclast differentiation. These findings identify a key role for Sirt6 in promoting RANKL-induced osteoclastogenesis and provide further insight into the mechanisms in fine-tuning the transcriptional regulatory network for osteoclastogenesis.

## Results

### Increased bone mass in *Sirt6* ^*f l/f l*^ *Mx1Cre* mice

**Figure 1:**
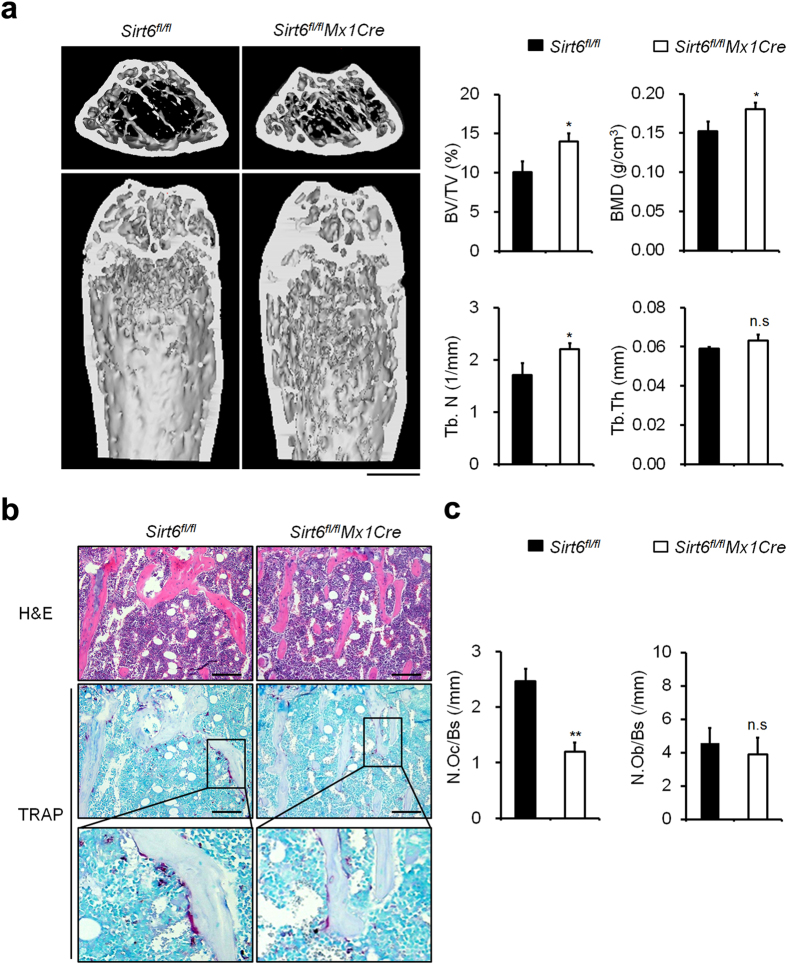
*Sirt6 *^fl/fl^*Mx1Cre* mice exhibited a high bone mass phenotype. (**a**) Microcomputed tomography (μCT) analysis of the femurs of 16-week-old *Sirt6*^*fl/fl*^(n = 5) and *Sirt6*^*fl/fl*^*Mx1Cre* (n = 5) male mice. BV/TV, bone volume per tissue volume; Tb.N, trabecular number; BMD, bone mineral density; Tb.Th, trabecular thickness. Scale bar, 1 mm. **P < *0.01.* (**b**) Histological analysis of the femurs from 16-week-old *Sirt6*^*fl/fl*^and *Sirt6*^*fl/fl*^*Mx1Cre* mice. Histology sections were stained with hematoxylin and eosin (upper) and TRAP (middle). Magnified images of the boxed area are shown (bottom). Scale bar, 50 μm. (**c**) Quantitative histological analysis of parameters in *Sirt6*^*fl/fl*^and *Sirt6*^*fl/fl*^*Mx1Cre* mice (n = 5); N.Oc/BS, osteoclast number per bone surface; N.Ob/BS, osteoblast per bone surface. (a,c) *P < 0.01, **P < *0.05.* n.s: not significant. Data are represented as mean ± S.D.

Global loss of Sirt6 expression in mice leads to premature death between 3 and 4 weeks of age after birth^19^. Moreover, myeloid-specific deletion of Sirt6 using *LysMCre* transgenic mice was shown to have profound liver inflammation^24^. To assess skeletal phenotypes following *Sirt6* deletion in adult stage of mice, we examined Sirt6 conditional knockout mice by crossing *Sirt6*^*flox/flox*^ mice (*Sirt6*^*fl/fl*^) with inducible *Cre* system, *Mx1Cre* mice instead of *LysMCre* or *CtsKCre* mice. In the *Sirt6*^*fl/fl*^*Mx1Cre* mice, the *Sirt6* gene is deleted upon polyinosinic-polycytidylic acid (poly I:C) treatment in osteoclast precursors, which allowed us to examine the effect of Sirt6 depletion on osteoclast formation. We first analyzed the bone phenotype of *Sirt6*^*fl/fl*^*Mx1Cre* mice at the age of 16 weeks, which had received polyI:C injection at the age of 10 d. The bone volume, the trabecular numbers, and bone mineral density were significantly increased in the *Sirt6*^*fl/fl*^*Mx1Cre* mice, without any change in the trabecular thickness (Fig. 1a). Bone morphometric analysis indicated a decrease in the osteoclast number of cells (Fig. 1b,c), but the osteoblast number was not changed in the *Sirt6*^*fl/fl*^*Mx1Cre* mice. These results suggested that Sirt6 in osteoclast precursor cells positively regulated osteoclast numbers *in vivo*.

### Impaired osteoclastogenesis in *Sirt6*-deficient cells

**Figure 2:**
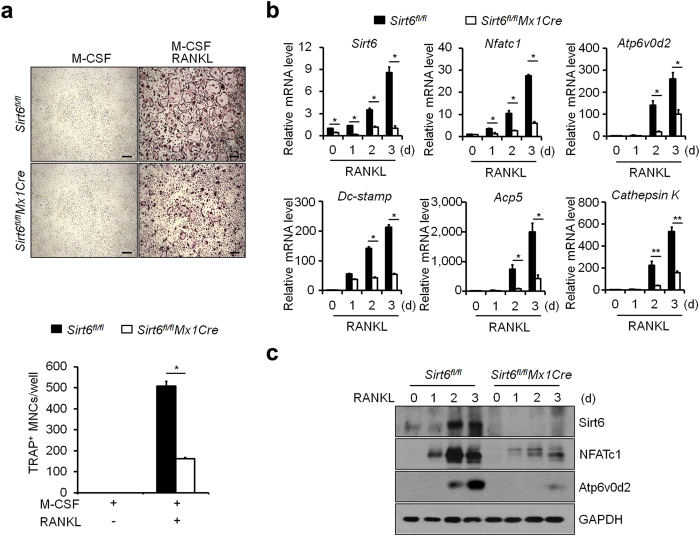
Sirt6-deficient BMMs impaired osteoclast differentiation. (**a**) BMMs from 6 week-old *Sirt6*^*fl/fl*^ and *Sirt6*^*fl/fl*^*Mx1Cre* mice were cultured in the presence of M-CSF (30 ng/ml) and RANKL (100 ng/ml) for 5 days and stained with TRAP. Number of TRAP^+^ MNCs (>5 nuclei) was counted as osteoclasts. Scale bar, 200 μm. *P < *0.01.* Data are represented as mean ± S.D. (**b**) Quantitative real-time PCR analysis of *Sirt6*, *Nfatc1*, and *Atp6v0d2* mRNAs in *Sirt6*^*fl/fl*^ and *Sirt6*^*fl/fl*^*Mx1Cre* BMMs stimulated with RANKL. *P < 0.05, **P < *0.01.* Data are represented as mean ± S.D. (**c**) As in (**b**), except that cell lysates were subjected to immunoblot analysis as indicated. GAPDH was used as a loading control.

*In vitro* osteoclast differentiation of bone marrow-derived monocyte/macrophage precursor cells (BMMs) derived from *Sirt6*^*fl/fl*^*Mx1Cre* mice was investigated by measuring the number of multinucleated cells (MNCs) positive for the osteoclast marker tartrate-resistant acid phosphatase (TRAP^+^) after stimulation of with RANKL in the presence of M-CSF. The number of TRAP^+^ MNCs was markedly decreased in the *Sirt6*^*fl/fl*^*Mx1Cre* cells compared with the control cells (Fig. 2a and Supplementary Fig. S1). Further, TRAP staining showed that a decrease in osteoclast size and in the number of nuclei per osteoclast was observed in marrow cultures from *Sirt6*^*fl/fl*^*Mx1Cre* mice compared with wild-type cultures, suggesting that Sirt6 regulates the fusion of osteoclast precursors as well as the formation of mature osteoclasts. In *Sirt6*^*fl/fl*^*Mx1Cre* cells, the expression of *Nfatc1* and its target genes, including *Atp6v0d2, Dc-stamp, Acp5, and Cathepsin K* was decreased at the mRNA and/or protein levels (Fig. 2b,c). However, there was no difference in bone resorbing activity in *Sirt6*^*fl/fl*^*Mx1Cre* osteoclasts when the same number of mature osteoclasts were seeded (Supplementary Fig. S2), suggesting that the increase in bone volume in the *Sirt6*^*fl/fl*^*Mx1Cre* mice was caused by the decreased number of osteoclasts, not by a decrease in their activity.

**Figure 3:**
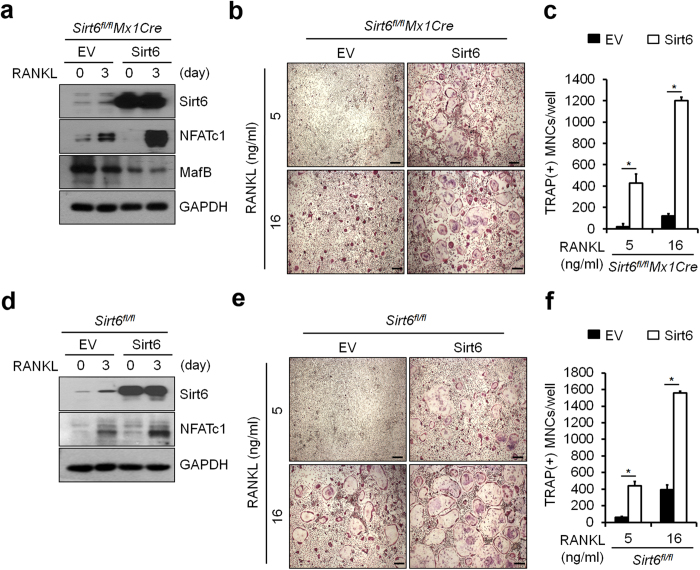
Sirt6 positively regulated osteoclastogenesis. (**a**) BMMs from *Sirt6*^*fl/fl*^*Mx1Cre* mice were transduced with pMX-puro (control, EV) or Flag-tagged Sirt6 retrovirus by stimulation with RANKL (50 ng/ml) for 3 days in the presence of M-CSF. Protein lysates were subjected to immunoblot analysis with Sirt6 and NFATc1 antibody. (**b**) Transduced BMMs were stained for TRAP after 5 days. Scale bar, 200 μm. (**c**) Number of TRAP^+^ MNCs (>5 nuclei) was counted as osteoclasts. (**d**) As in (**a**), except that BMMs from *Sirt6*^*fl/fl*^ mice were used. Protein lysates were subjected to as in (**a**). (**e**) Transduced BMMs were stained as in (**b**). Scale bar, 200 μm. (**f**) Number of TRAP^+^ MNCs (>5 nuclei) was counted as in (**c**). GAPDH was used as a loading control. *P < *0.05.* Data are represented as mean ± S.D.

To ensure that the observed *Sirt6*^*fl/fl*^*Mx1Cre* BMMs phenotype is solely a result of *Sirt6* deficiency, we determined whether impaired osteoclastogenesis in *Sirt6*^*fl/fl*^*Mx1Cre* BMMs could be rescued by reintroduction of *Sirt6*. BMMs from *Sirt6*^*fl/fl*^and *Sirt6*^*fl/fl*^*Mx1Cre* were infected with a Sirt6-expressing retrovirus or control virus. Expression of Sirt6 protein was confirmed by immunoblotting (Fig. 3a). As expected, re-expression of Sirt6 restored the ability of *Sirt6*^*fl/fl*^*Mx1Cre* BMMs to differentiate into mature osteoclasts in the presence of RANKL (Fig. 3b,c), indicating that the *Sirt6*^−/−^ phenotype only resulted from the null mutation of *Sirt6*. Consistently, ectopic expression of Sirt6 increased the sensitivity of osteoclast differentiation to RANKL signaling in osteoclast precursor cells (Fig. 3d–f). Of note, enhanced osteoclastogenesis was observed in lower concentrations of RANKL (5–16 ng/ml) compared to the concentration of RANKL (100 ng/ml) used for Fig. 2a. This may be due to effect of Sirt6 overexpression, which may cause enhanced osteoclast formation in the lower concentrations of RANKL. It is noteworthy that the expression of NFATc1 was accelerated by Sirt6 overexpression in the presence of RANKL (Fig. 3a,d). These results indicate that Sirt6 deletion in osteoclast precursor cells results in decreased osteoclast differentiation through down-regulation of NFATc1 levels.

To exclude the possibility that impaired osteoclastogenesis in *Sirt6*^*fl/fl*^*Mx1Cre* BMMs was due to decreased numbers of osteoclast precursors derived from the hematopoietic lineage, we examined the ratio of the osteoclast precursor cells among the bone marrow cells. The percentage of the most highly osteoclastogenic c-kit^+^c-fms^+^ cells in the CD11b^lo/−^CD3ε^−^B220^−^ population^25^ was similar between the control and *Sirt6*^*fl/fl*^*Mx1Cre* mice, indicating that the proportion of osteoclast precursor cells in the bone marrow was unchanged (Supplementary Fig. S3a). In addition, there was no significant difference in the proliferation rate of CD11b^+^ cells cultured in the presence of M-CSF for 2 d (Supplementary Fig. S3b).

### Sirt6 is a target of the NFATc1

**Figure 4:**
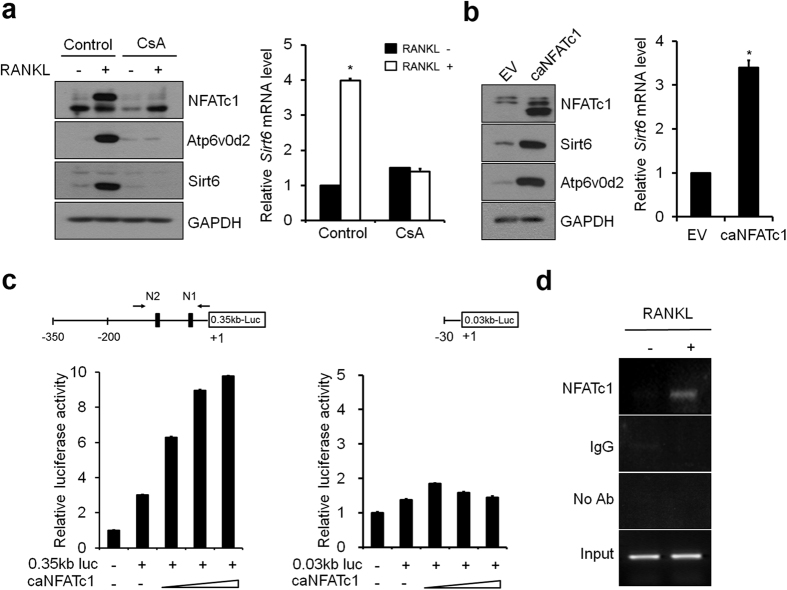
NFATc1 regulated Sirt6 expression during osteoclastogenesis. (**a**) (*Left*) BMMs were cultured with DMSO (control) or cyclosporine A (CsA, 10 μM) in the absence or presence of RANKL for 3 days and subjected to immunoblot analysis with Sirt6, NFATc1 and Atp6v0d2 antibody. GAPDH was used as a loading control. (*Right*) Quantitative real-time PCR was performed to detect expression of the Sirt6. *P < *0.01.* Data are represented as mean ± S.D. (**b**) (*Left*) BMMs infected with pMX-puro (EV, empty vector) or constitutively active NFATc1 (caNFATc1) retroviruses were cultured for 6 days with M-CSF alone, and then cell lysates were subjected to immunoblot analysis with Sirt6, NFATc1 and Atp6v0d2 antibody. GAPDH was used as a loading control. (*Right*) Quantitative real-time PCR was performed to detect expression of the Sirt6. *P < *0.01.* Data are represented as mean ± S.D. (**c**) Schematic representation of *Sirt6* promoter luciferase reporters, which have different sizes, is shown. Black box indicates two putative NFATc1-binding sites (N1 and N2). +1 indicates the transcription start sites. *Sirt6* promoter luciferase reporter vectors (0.35 kb-Luc and 0.03 kb-Luc) were transfected into RAW 264.7 cells with increasing concentrations caNFATc1(100, 200 and 300 ng). Data are presented as the mean ± S.D. (**d**) Recruitment of NFATc1 to the *Sirt6* promoter in the presence or absence of RANKL was detected by ChIP assay. Samples were subjected to PCR with N1 and N2 specific primers for the NFATc1 binding sites of the *Sirt6* promoter region.

Since Sirt6 was only slightly expressed in BMMs, but was markedly induced by RANKL but not by lipopolysaccharide (Supplementary Fig. S4), we examined whether NFATc1 regulates Sirt6 expression during osteoclastogenesis. It has been shown that cyclosporin A (CsA), an inhibitor of calcineurin activity, inhibits RANKL-mediated osteoclastogenesis by suppressing *Nfatc1* gene expression^26^. RANKL-dependent induction of Sirt6 at both the protein and mRNA levels was markedly decreased by CsA-mediated NFATc1 inhibition (Fig. 4a). Conversely, we examined whether overexpression of a constitutively active form of NFATc1 (caNFATc1) in BMMs affected the expression of Sirt6. Sirt6 levels were up-regulated by transduction of ca-NFATc1 alone, even without RANKL stimulation (Fig. 4b). These observations suggested that *Sirt6* gene is specifically induced by RANKL in osteoclast precursors through NFATc1. The 0.35-kb *Sirt6* promoter fragment (–350 to +1), linked to luciferase reporter construct, was activated in response to NFATc1 expression (Fig. 4c). However, luciferase activities were completely abolished in the Sirt6 0.03-kb construct (–30 to +1) as compared with the 0.35-kb *Sirt6* promoter fragment. Consistently, two putative NFAT-binding DNA element^27^ were present in the 5′-flanking region of *Sirt6* (Supplementary Fig. S5). Chromatin immunoprecipitation assays indicated that binding of NFATc1 to the 5′-flanking sequence of *Sirt6* promoter increased during osteoclast differentiation (Fig. 4d). Together, these data indicate that Sirt6 is a direct transcriptional target of NFATc1 during osteoclastogenesis.

### Sirt6 reciprocally regulates Blimp1 and MafB expression

**Figure 5:**
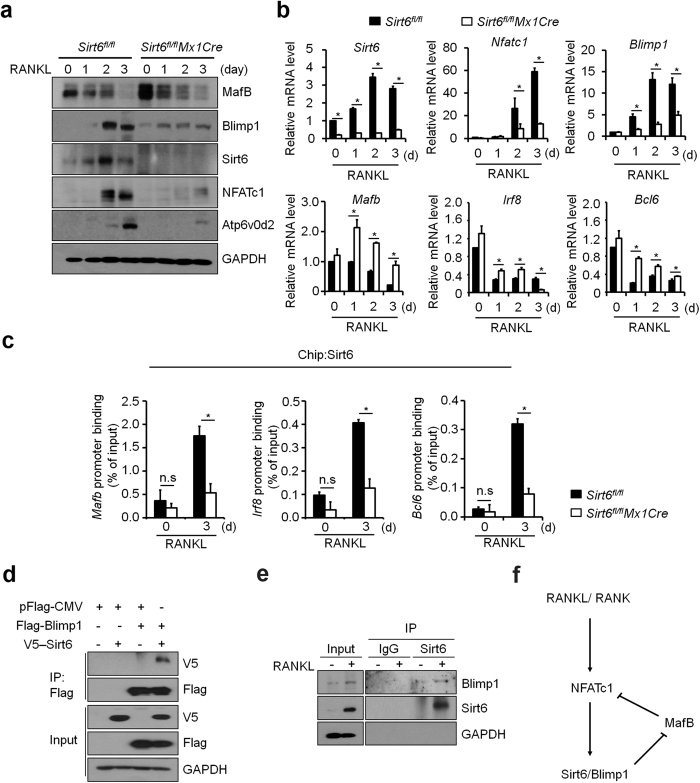
MafB expression was enhanced in Sirt6-deficient BMMs. (**a**) *Sirt6*^*fl/fl*^and *Sirt6*^*fl/fl*^*Mx1Cre* BMMs were cultured with M-CSF (30 ng/ml) and RANKL (100 ng/ml) for the indicated time periods. Cell lysates were subjected to immunoblot analysis with MafB, Blimp1, Sirt6, NFATc1 and Atp6v0d2 antibody. GAPDH was used as a loading control. (**b**) Quantitative real-time PCR was performed for the mRNA expression of *Sirt6* and osteoclastogenic genes, such as *Nfatc1*, *Atp6v0d2,* and *Blimp1,* and anti-osteoclastogenic genes, such as Mafb, Irf8 and Bcl6. *P < *0.01.* Data are represented as mean ± S.D. (**c**) Recruitment of Sirt6 to promoters of anti-osteoclastogenic genes such as *Mafb*, *Irf8* and *Bcl6* in the presence or absence of RANKL was detected by ChIP assay. Samples were subjected to quantitative real-time PCR with specific primers for the Sirt6-binding sites in the *Mafb*, *Irf8* and *Bcl6* promoter region. Sirt6 occupancy at the promoter is shown relative to the background signal with IgG control antibody. *P < *0.01.* n.s: not significant. Data are represented as mean ± S.D. (**d**) 293 T cells were transfected with Flag-tagged Blimp1 together with V5-tagged Sirt6 construct. Protein lysates were prepared and subjected to immunoprecipitation (IP) using FLAG antibody. The total amount of transfected DNA was kept constant by addition of empty pFlag-CMV expression vector. (**e**) BMMs were cultured with M-CSF (30 ng/ml) and RANKL (100 ng/ml) for 3 days. Protein lysates were prepared and subjected to co-immunoprecipitation using the anti-Sirt6 or control IgG antibodies. (**f**) Working model for the role of Sirt6 during osteoclastogenesis. During osteoclastogenesis, Sirt6 was induced by the RANKL-NFATc1 axis. Sirt6 in cooperation with Blimp1 suppressed anti-osteoclastogenic transcription factor such as MafB.

Deficiency of Sirt6 did not affect activation of signaling cascades consisting of MAPKs (p38, ERK, and JNK) and Akt stimulated by M-CSF or RANKL in BMMs (Supplementary Fig. S6). Although it has been reported previously that Sirt6 functions as a transcriptional repressor in other cell types^22^,^23^,^28^, transcription factors can function as either a positive or a negative transcriptional regulator in a context-dependent manner^29^. To investigate whether Sirt6 functions as a transcriptional regulator during osteoclastogenesis, we examined expression of *Blimp1* and *Mafb* which were shown to function as a positive- and a negative-regulator of osteoclastogenesis, respectively^12^,^30^. Sirt6 deficiency increased the expression of *Mafb* significantly with a concomitant decrease in Blimp1 expression at protein and mRNA levels (Fig. 5a,b). *Irf8* and *Bcl6* expression in *Sirt6*^*fl/fl*^*Mx1Cre* BMMs also increased upon RANKL stimulation. We analyzed whether Sirt6 binds to the promoters of *Mafb*, *Irf8*, and *Bcl6* genes and observed more obvious occupancy of Sirt6 in the promoters in wild-type cells in comparison with *Sirt6*^*fl/fl*^*Mx1Cre* cells (Fig. 5c). Furthermore, Sirt6 interacted with Blimp1 in mammalian cells (Fig. 5d) as well as in RANKL-stimulated osteoclast precursors (Fig. 5e).

These results suggest that Sirt6 cooperates with Blimp1, which in turn regulates expression of transcriptional repressors of osteoclastogenesis, such as *Mafb* (Fig. 5f).

## Discussion

Previous studies that were based on global ablation of Sirt6 outlined an important role for Sirt6 in bone homeostasis. Histomorphometric analysis of bone and the bone cell biology of *Sirt6*^−/−^ mice revealed that deficiency of *Sirt6* caused osteopenia due to mainly impaired function of osteoblasts^19^,^23^. These studies examined mice at 3 weeks and provided important evidence for the function of Sirt6 in specifying osteoblast differentiation during development. However, the requirement for Sirt6 during osteoclast differentiation in adult skeletal remodeling remained unresolved. Here *Mx1-Cre* was used to delete *Sirt6* in mice at 10 days of age as was previously done to investigate the role of NFATc1 during osteoclastogenesis^31^. We identified a new role of Sirt6 as a key positive regulator of osteoclastogenesis. Sirt6 deficiency in osteoclast precursors inhibited osteoclastogenesis by suppressing expression of the key transcription regulator NFATc1 and Blimp1, and by augmenting expression of the transcriptional repressor MafB, which prevented induction of the NFATc1-mediated osteoclast differentiation program.

The balance between positive- and negative-regulation of osteoclastogenesis is important for bone homeostasis and in order to prevent excessive bone resorption in inflammatory and other diseases. Positive signaling pathways and transcription factors that promote osteoclastogenesis have been extensively studied and are well characterized^2^,^32^. A typical example is NFATc1, whose activity and expression are maintained at an extremely high level by RANKL stimulation, thereby promoting osteoclastogenesis. Recently, it has been known that osteoclastogenesis is also negatively regulated by a number of transcriptional repressors, including MafB, IRF-8, and Bcl6^11^,^30^,^33^,^34^,^35^. These factors suppress transcription of *Nfatc1* and its target genes, and their expression is downregulated during osteoclastogenesis to allow the gene expression program associated with osteoclast differentiation to proceed. Interestingly, *Sirt6*^*fl/fl*^*Mx1Cre* BMMs formed significantly decreased numbers of osteoclasts *in vitro* and have decreased nuclei per osteoclast. The decreased fusion of osteoclast precursors most likely reflects the reduced expression of Atp6v0d2 and DC-STAMP in osteoclast precursors from *Sirt6*^*fl/fl*^*Mx1Cre* BMMs. Similarly, a previous report also has shown that NFATc1 induces osteoclast fusion via upregulation of Atp6v0d2 and DC-STAMP^9^.

How is the balance between positive- and negative-regulation achieved and maintained within transcriptional network? To this end, a signaling pathway usually may stimulate the negative-feedback regulatory pathways to keep in check any excesses in the cell differentiation program. In this context, the fact that Sirt6 was induced by the RANKL-NFATc1 axis and was involved in the negative regulation of anti-osteoclastogenic gene expression places Sirt6 in a transcriptional regulatory network of osteoclastogenesis. A previous study indicated that Sirt6 interacts with the NF-κB RelA subunit and deacetylates H3K9 at NF-κB target gene promoters and the loss of Sirt6 caused activation of NF-κB dependent gene expression^23^. Sirt6 has also been well characterized as a co-repressor of the transcription factor HIF1α to control the expression of multiple glycolytic genes^22^ and c-Jun to inhibit pro-inflammatory gene expression^28^. It remains to be determined how Sirt6 exerts negative effects of anti-osteoclastogenic gene expression. Recently, Blimp1 has been placed upstream of several repressors of osteoclastogenesis, including MafB, IRF8, and Bcl6 during osteoclastogenesis^11^,^30^,^35^. An increase in Blimp1 expression after RANKL stimulation serves to down-regulate expression of repressors of osteoclastogenesis^10^,^12^. The function of Sirt6 in osteoclasts is similar to the role of Blimp1, in that they are induced by the RANKL-NFATc1 axis and repress the genes involved in anti-osteoclastogenesis. In addition, Sirt6 binds to promoters of *Mafb*, *Irf8*, and *Bcl6* genes. Because Sirt6 interacts with Blimp1 in osteoclast precursors, it is reasonable to hypothesize that Sirt6 in cooperation with Blimp1 serves to switch-off the brakes in osteoclastogenesis by acting as a negative regulator of anti-osteoclastogenic gene expression (Fig. 5f). Further understanding of the gene regulatory programs mediated by Sirt6-Blimp1 axis may provide a novel molecular basis for therapeutic strategies against bone and joint diseases.

## Methods

### Reagents and plasmids

Recombinant human M-CSF was purchased from R&D Systems (Minneapolis MN, USA). RANKL was obtained from PeproTech EC (London, UK). CsA was purchased from Calbiochem (La Jolla CA, USA) and poly I:C was from Sigma-Aldrich (St. Louis MO, USA). Primary antibodies used in the study included monoclonal anti-FLAG, anti-Sirt6 (Sigma-Aldrich), anti-Blimp1 (Cell Signaling Technology, Beverly MA, USA), anti-V5 (Invitrogen, Carlsbad CA, USA), anti-MafB (Novus Biologicals, Littleton CO, USA), anti-NFATc1 and anti-GAPDH (Santa Cruz Biotechnology Inc., Santa Cruz CA, USA) followed by secondary horseradish peroxidase-conjugated antibody. Anti-Atp6v0d2^36^ antibody was kindly provided by Y. Choi (University of Pennsylvania, Philadelphia PA, USA). The pCDH-3x-Flag-Sirt6 and pcDNA 3.1-V5-Sirt6 were made as described previously^21^. For retroviral expression, the Flag-Sirt6 DNA was subcloned into pMX-puro to make pMX-puro-Flag-Sirt6. A retroviral vector, pMX-puro was provided by Dr T Kitamura (University of Tokyo, Tokyo, Japan). The pMX-puro-Flag-Blimp1 plasmid^12^ was provided by J. Rho (Chungnam National University, Daejeon, Korea). The retroviral vector containing a constitutively active form of NFATc1 (caNFATc1) was previously described^37^.

### Primary cells and cell line

BMMs were obtained from murine bone marrow precursors of 4- to 6-week-old C57BL/6 mice (The Jackson Laboratory, Bar Harbor ME, USA) as described^38^. BMMs were cultured for 3 days in α-minimum essential medium (α-MEM; HyClone, South Logan UT, USA) supplemented with 10% fetal bovine serum (FBS; HyClone) and antibiotics containing M-CSF (30 ng/ml). After 3 days, the non-adherent cells were removed and adherent cells (BMMs) were harvested to obtain osteoclast precursor cells of the monocyte/ macrophage lineage. 293 T cells and RAW 264.7 cells were cultured in Dulbecco’s modified Eagle’s medium (DMEM; HyClone) supplemented with 10% FBS with antibiotics. PLAT-E cells were cultured in DMEM with 10% FBS and antibiotics containing blasticidin (10 mg/ml) (Invitrogen) and puromycin (1 mg/ml) (Sigma-Aldrich). PLAT-E cells were provided by Dr T. Kitamura (University of Tokyo).

### *In vitro* osteoclast differentiation

Osteoclasts were prepared form bone marrow cells using a standard method^39^. In brief, the precursor cells were cultured for 3 days with M-CSF (30 ng/ml) and RANKL (100 ng/ml) for osteoclast differentiation. The cells were stained with TRAP staining kit (Sigma-Aldrich). TRAP positive multinucleated (>5 nuclei) cells (MNCs) were counted as osteoclast-like cells. TRAP assays were also carried out as previously described^38^. Data are presented as the averages of 3 separate experiments done in triplicate ± S.D.

### Mice

*Sirt6*^*fl/fl*^ mice were generated as described^24^. To obtain Sirt6 conditional knock-out mice (*Sirt6*^*fl/fl*^*Mx1Cre*) in hematopoietic cells, homozygous *Sirt6*^*fl/fl*^ mice were crossed with *Mx1Cre* transgenic mice [Tg(Mx1-cre)1Cgn] purchased from Jackson Laboratory^40^. For induced expression of Cre in *Mx1Cre* mouse, male mice (10 days after birth) were injected three times intraperitoneally with 250 μg poly I:C/20 g body mass every other day for 6 days to generate *Sirt6*^*fl/fl*^*Mx1Cre* mice. Mice, 6 weeks old after the first injection, were analyzed for *in vitro* studies, whereas 16 weeks old mice were analyzed for *in vivo* experiments. All experiments were approved by the Institutional Animal Care and Use Committee of Ewha Laboratory Animal Genomics Center, and were carried out in accordance with the approved guidelines.

### Retroviral infection

PLAT-E retrovirus packaging cell was transfected with pMX-puro empty, Sirt6-Flag, or caNFATc1 retroviral vector using polyethylenimine (Sigma-Aldrich) reagent and the supernatant was collected 48 hours after transfection. BMMs were infected with the supernatant including retroviruses in the presence of M-CSF (30 ng/ml) and polybrene (10 μg/ml) for 6 hours as previously described^41^. After 24 hours infection, to select for infected cells, media was changed in presence of M-CSF (30 ng/ml) and puromycin (2 μg/ml) for 2 days. Puromycin-resistant BMMs were used for osteoclast differentiation in the presence of M-CSF (30 ng/ml) and RANKL (100 ng/ml) for an additional 3–5 days.

### Quantitative real time PCR

Total RNA from cells was extracted from using the TRIzol (Invitrogen). cDNA were synthesized with oligo (dT) primers and M-MLV reverse transcriptase (SolGent, Seoul, Korea). Real-time quantitative PCR was performed in triplicate on ABI PRISM 7300 unit (Applied Biosystems, Foster City CA, USA) and the SYBR Green Master kit (Kapa Biosystems, Wilmington MA, USA). Amounts of mRNAs were normalized relative to actin mRNA. Primers specific for murine *Sirt6*, *Nfatc1*, *Atp6V0d2, Dc-stamp, Acp5, Catepsin K, Blimp1, Mafb, Bcl6, Irf8* and *Actin* were used. The following primers were used; *Sirt6* sense 5′-CATGGGCTTCCTCAGC-3′ and antisense 5′-AACGAGTCCTCCCAGT-3′; *Nfatc1* sense 5′-CCAGAAAATAACATGC-3′ and antisense 5′-GTGGGATGTGAACTCG-3′; *Atp6v0d2* sense 5′-CAGAGATGGAAGCTGT-3′ and antisense 5′-TGCCAAATGAGTT CAG-3′; *Dc-stamp* sense 5′-TGGAAGTTCACTTGAAACTACGTG-3′ and antisence 5′-CTCGGTTTCCCGTCAGCCTCTCTC; *Acp5* sense 5′-CCATTGTTAGCCACATAC ATACGG-3′ and antisense 5′-ACTCAGCACATAGCCCACAC-3′; *Cathepsin K* sense 5′-ACGGAGGCATTGACTCTGAAGATG-3′ and antisense 5′-CTGCATGGTTCACA TATCACGGTC-3′; *Blimp1* sense 5′-TTCTTGTGTGGTATTG-3′ and antisense 5′-TTGGGGACACTCTTTG-3′; *Mafb* sense 5′-AGTGTGGAGGACCGCTT-3′ and antisense 5′-CAGAAAGAACTCAGGAG-3′; *Bcl6* sense 5′-AGACGCACAGTGACA AA-3′ and antisense 5′-GCTCCACAAATGTTACA-3′; *Irf8* sense 5′-GATCGAACAG ATCGACA-3′ and antisense 5′-CTGGGCTCTTGTTCAGA-3′; *Actin* sense 5′-GCTT CTTCTTTGCAGCTCCT-3′ and 5′-ATCGTCATCCATGGCGA-3′.

### Luciferase reporter assay

Luciferase reporter assay was performed as previously described^42^. Briefly, RAW 264.7 cells were co-transfected with pGL3 control reporter, pGL3-0.35 kb and pGL3-0.03 kb Sirt6 luciferase reporter constructs and various amounts of caNFATc1 (100, 200 and 300 ng) using LTX Reagent (Invitrogen) according to the manufacturer’s instructions. After 36 hours, luciferase activity was measured using the dual-luciferase reporter assay system (Promega, Madison WI, USA) according to the manufacturer’s instructions. Luciferase activity was measured in triplicate and normalized the activity of the control (pRenilla).

### Chromatin immunoprecipitation (ChIP) analysis

ChIP assay was performed with an EZ-ChIP kit (Millipore, Bedford MA, USA) according to the manufacturer’s instructions. In brief, BMMs (2 × 10^6^ cells on 10 cm) were cultured in presence of M-CSF (30 ng/ml) with or without RANKL (100 ng/ml) for 3 days. After 3 days, cells were fixed with formaldehyde. Cells were washed twice using cold PBS containing protease inhibitors, centrifuged and suspended in 200 μl of SDS lysis buffer containing protease inhibitors for 10 minutes on ice. Lysates were sonicated to reduce DNA length to between 200 and 1000 base pairs and centrifuged. Supernatant fraction was diluted in ChIP dilution buffer containing protease inhibitors. The chromatin solution was precleared with salmon sperm DNA/protein agarose slurry for 30 minutes at 4 °C with rotation. After preclearing, the supernatant was used for ChIP with anti-NFATc1 (7A6), anti-Sirt6 (Sigma-Aldrich) or control IgG (Santa Cruz) for control overnight at 4 °C with rotation. Final immunoprecipitated DNA was analyzed by PCR. *Sirt6* promoter (N1N2) primer was generated to detect DNA segments located near the NFATc1 binding site N1 at −16/−11(GGAAA) and NFATc1 binding site N2 at −138/−133 (GGAAA). PCR was performed using specific primers for 35 cycles. PCR products were subjected to 1.5% agarose gels electrophoresis and visualized with ultraviolet light. The PCR primers were used; *Sirt6* promoter N1N2 sense 5′-CAGGACTGGGGAATCCACTA-3′ and antisense 5′-CGACAACCCTGCTGCATAAT-3′. *Mafb* promoter sense 5′-CCTTGCCTTGTCCT GAAG-3′ and antisense 5′-GAGGGGGTATGAAGGAGAGG-3′; *Irf8* promoter sense 5′-TCCCTCCCTCCTTCTCCTTA-3′ and antisense 5′-AAGCCCTGAGTGCACAGACT-3′; *Bcl6* promoter sense 5′-CAGCCACCCTGAGTTTACAA-3′ and antisense 5′-CGTTCCAGCACTGTTTTGAA-3′.

### Immunoprecipitation and immunoblot analysis

293 T cells were transiently transfected with V5-Sirt6 and Flag-Blimp1 using polyethylenimine reagent. Cells were washed twice with cold-PBS and lysed in RIPA buffer (10 mM Tris-HCl (pH 8.0), 150 mM NaCl, 1% NP-40, 1 mM EDTA, 0.2% sodium deoxycholate) supplemented with protease inhibitors. After incubation for 1 hour on ice, lysates were centrifuged at 14,000 rpm for 20 minutes at 4 °C. Subsequently, protein concentration was measured by Bradford assay (Bio-Rad, Hercules CA, USA). Equivalent amounts of protein were incubated with anti-Flag antibodies overnight at 4 °C, followed by an incubation with protein A agarose beads (Millipore). The beads were washed five times with a washing RIPA buffer containing protease inhibitors, resuspended with 2X sample loading buffer, and immunocomplexes were resolved by SDS-PAGE and analyzed by immunoblot with antibodies.

### Bone histomorphometry and microcomputed tomography (μCT) analysis

*Sirt6*^*fl/fl*^ and *Sirt6*^*fl/fl*^*Mx1Cre* mice were fixed in 10% formaldehyde for 24 hours, decalcified in 0.5 M EDTA (pH 7.4) for 14 days, and embedded in paraffin, and then cut into 5 μm sections. Hematoxylin and eosin (H&E) or TRAP staining were also carried out according to a standard protocol^36^. The measurement of osteoclast number (Oc.N/BS, mm) and osteoblast number (Ob.N/BS, mm) was performed at tibial metaphyseal cancellous bone areas just below the growth plate-metaphyseal junction with an image-analyzing system (*i*MT image analysis software, *i*MTechnology, Daejeon, Korea) linked to a light microscope (Olympus, Tokyo, Japan). Quantitative μCT was performed with SkyScan 1076 μCT scanner system (SkyScan, Kontich, Belgium). The data from scanned slices were used for the three-dimensional analysis to calculate femoral morphometric parameters by CT-AN 1.10 (SkyScan). The measurements, terminology, and units for both bone histomorphometry and μCT analysis were expressed according to recommendations by the Nomenclature Committee of the American Society of Bone and Mineral Research^43^. Bone histomorphometry and μCT were performed on mouse long bones (femurs).

### Statistics

Data were expressed as mean ± S.D. from at least three independent experiments. Statistical differences were analyzed by two-tailed student’s *t*-test. *P* < 0.05 was considered statistically significant.

## Additional Information

**How to cite this article**: Park, S. J. *et al.* Sirt6 cooperates with Blimp1 to positively regulate osteoclast differentiation. *Sci. Rep.* **6**, 26186; doi: 10.1038/srep26186 (2016).

## Supplementary Material

Supplementary Information
