# Supplementary material for: Sirt6 cooperates with Blimp1 to positively regulate osteoclast differentiation
Source: Sci Rep. 2016 May 18;6:26186. doi: 10.1038/srep26186 (PMC4870620; doi:10.1038/srep26186)
Supplement: Supplementary Information [file srep26186-s1.pdf]

Supplementary Information

Sirt6 cooperates with Blimp1 to positively regulate osteoclast differentiation

So Jeong Park[1,2], Jeong-Eun Huh[1,2], Jihye Shin[1,2], Doo Ri Park[1,2], Ryeojin Ko[1,2], Gyu-Rin Jin[2,5], Dong-Hyun Seo[3], Han-Sung Kim[3], Hong-In Shin[4], Goo Taeg Oh[1], Hyun Seok Kim[2,5,*], and Soo Young Lee[1,2,*]

[1]Department of Life Science, Ewha Womans University, Seoul 120-750, Korea.
[2]The Research Center for Cellular Homeostasis, Ewha Womans University, Seoul 120-750, Korea
[3]Department of Biomedical Engineering, College of Health Science, Institute of Medical Engineering, Yonsei University, Wonju, Korea.
[4]IHBR, Department of Oral Pathology, School of Dentistry, Kyungpook National University, Daegu 700-412, Korea.
[5]Department of Bioinspired Science, Ewha Womans University, Seoul 120-750, Korea.

[*]Correspondence should be addressed to S.Y.L. (leesy@ewha.ac.kr) or H.S.K. (kimhs0601@ewha.ac.kr)

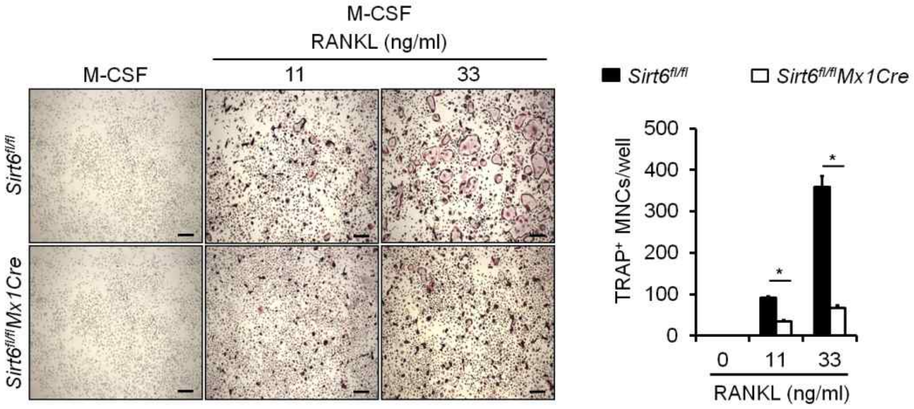

Supplementary Figure 1. Sirt6-deficient BMMs impaired osteoclast differentiation.

(a) BMMs from *Sirt6*$^{fl/fl}$ and *Sirt6*$^{fl/fl}$*Mx1Cre* mice were differentiated with M-CSF (30 ng/ml) and lower concentrations of RANKL (11-33 ng/ml), respectively and stained with TRAP. (b) Number of TRAP^{+} MNCs (>5 nuclei) was counted as osteoclasts. Scale bar, 200μm. *$P<0.01$. Data are presented as the mean ± S.D.

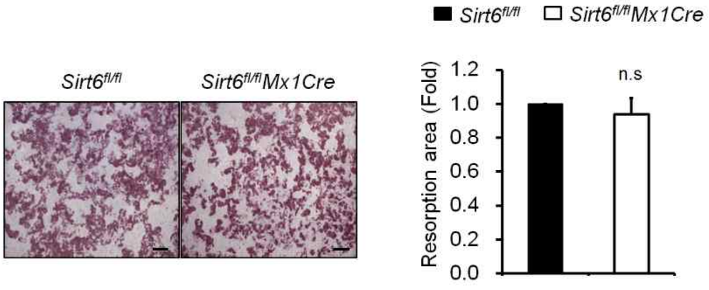

Supplementary Figure 2. Bone-resorbing activity of mature osteoclasts from *Sirt6$^{fl/fl}$* and *Sirt6$^{fl/fl}$Mx1Cre* BMMs. After differentiation of BMMs from *Sirt6$^{fl/fl}$* and *Sirt6$^{fl/fl}$Mx1Cre* mice into osteoclasts, the mature osteoclasts were harvested by trypsinization and cultured for 2 days on dentin slices. The resorbed dentin were stained with hematoxylin for visualization of pit formation. The area of resorption pits was measured with Image-Pro Plus 4.5 (Media Cyvernetics). Scale bar, 200μm n.s: not significant. Data are presented as the mean ± S.D.

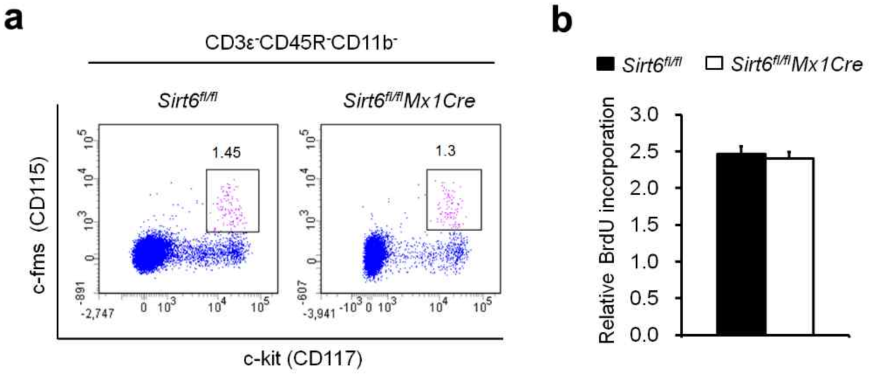

Supplementary Figure 3. Deficiency of Sirt6 did not change the proportion of osteoclast precursor cells in the bone marrow. (a) BMMs from *Sirt6*$^{fl/fl}$ and *Sirt6*$^{fl/fl}$*Mx1Cre* mice were stained with PerCP-Cyanine 5.5-conjugated CD11b, FITC-conjugated CD3ε, APC-conjugated c-kit (CD117), BV421-conjugated CD45R/B220 and PE-conjugated CD115 antibodies and analyzed with flow cytometry as described in Methods. The percentage of c-kit^{+}c-fms^{+} cells was calculated by the percentages of gated on the CD11b$^{lo/-}$CD3ε^{-}B220^{-} population. (b) BrdU staining. Proliferation was assessed by the absorbance of incorporated BrdU in *Sirt6*$^{fl/fl}$ and *Sirt6*$^{fl/fl}$*Mx1Cre* BMMs. Data are presented as the mean ± S.D.

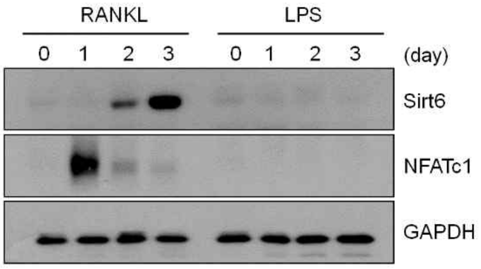

Supplementary Figure 4. Sirt6 expression was increased during osteoclastogenesis. BMMs were stimulated with RANKL (100 ng/ml) or LPS (100 ng/ml) for the indicated periods. Stimulated cells were lysed and subjected to immunoblot analysis with antibodies against Sirt6 and NFATc1. GAPDH was used as a loading control.

```
-472  gcgtggcttcgaactttctgtcccctcctgccagtgctataagcactccgggcagggctctacacactgagaactgtggatttttgtttt

-382  gctttgtttggtttcatttctgggttttgctgatgttgtctcttttgaaacagggagttggtctgggactcgcaatccccctgcctcgactcct

-288  gtgagtggcctcatcacttttctggctctggtcgcggatcccaggactggggaatccactaagtcagagaggtagtctcggccagc

-202  actgaagactaagcttgctgcatcaggagggcgcatgcgctctggaaacaatgctgtttttcacctccacgtacccaccccaccct
                                                NFATc1 binding site (N2)
-116  caggcacgtatctagggaaggcggctactctttcccgaccgtcccttcaccacttccggcggaagcggcctcaacaagggaaac
                                                                        NFATc1 binding site (N2)
 -32  tttattgttcccgtgcggcagcgccggcgacgATGTCGGTGAATTATGCAGCAGGGTTGTCGC

 +54  CTTACGCGGATAAGGGCAAGTGCGGGCTGCCCGAGGTAGAGCTGCAGTGTTCGAGTCA
```

Supplementary Figure 5. Nucleotide sequences of the putative promoter region of mouse *Sirt6*. The 0.5-kb *Sirt6* promoter contained two potential NFATc1-binding sites (N1 and N2). The consensus sequence of NFATc1 (5'-GGAAA-3') is marked red. The binding sites were evaluated by Genomatix software (https://www.genomatix.de/) program. The numbers on the left are the nucleotide positions relative to the transcription initiation site. The predicted transcription start region is indicated with a capital letter.

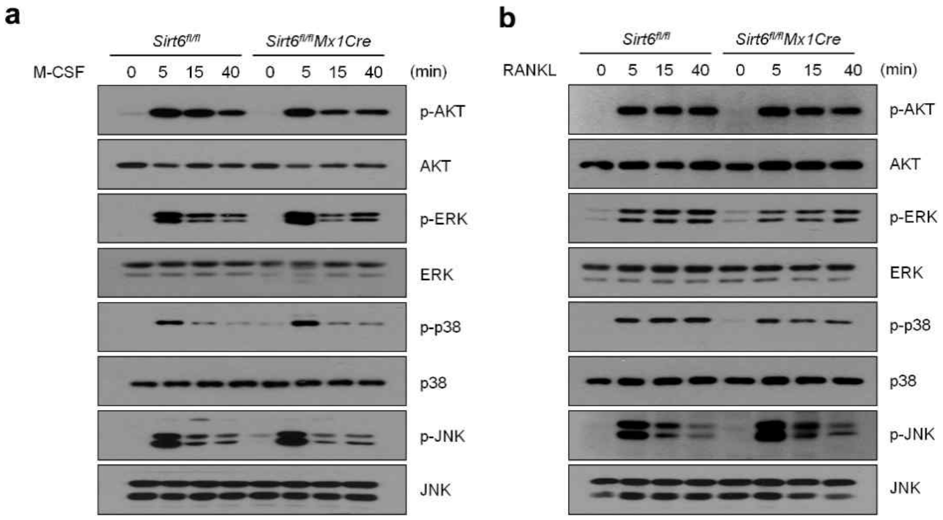

Supplementary Figure 6. Sirt6 deficiency does not affect RANKL- or M-CSF-mediated signaling. (a) Activation of Akt and MAPKs (ERK, p38, and JNK) in *Sirt6*$^{fl/fl}$ and *Sirt6*$^{fl/fl}$*Mx1Cre* BMMs in response to M-CSF (30 ng/ml). Total Akt, ERK, p38, and JNK served as the loading control. (b) As in (a), except that cells were stimulated by RANKL (100 ng/ml) as the indicated times.

Supplementary methods

Resorption pit assay.

BMMs from *Sirt6*$^{fl/fl}$ and *Sirt6*$^{fl/fl}$*Mx1Cre* mice were cultured for 2 days in complete medium (α-MEM) containing M-CSF (30 ng/ml) and RANKL (100 ng/ml). For the bone resorption assay, the osteoclasts generated in the presence of M-CSF (30 ng/ml) and RANKL (100 ng/ml) for 4 days were harvested by trypsinization and cultured for 2 days on dentin slices in the presence of M-CSF (30 ng/ml) and RANKL (100 ng/ml). Cells on the dentin slices were removed by washing with PBS, and stained with hematoxylin. Pit areas were photographed under a light microscope and analyzed using Image-Pro Plus program 4.5 (Media Cybernetics, Rockville, MD, USA). Dentin slices were provided by M. Takami (Showa University, Tokyo, Japan).

Flow cytometry. BMMs from 4- to 6 week-old *Sirt6*$^{fl/fl}$ and *Sirt6*$^{fl/fl}$*Mx1Cre* mice were cultured for 24 hours. After washing cells twice with phosphate buffer saline (PBS), cells were incubated with 0.5% BSA for 30 minutes. Cells were resuspended in 0.5 ml PBS/0.5% BSA, and then stained with the appropriate antibody for 1 hour. Cells surface fluorescence was determined using PerCP-Cyanine 5.5-conjugated CD11b, FITC-conjugated CD3ε, APC-conjugated c-kit (CD117), BV421-conjugated CD45R/B220 and PE-conjugated CD115 with appropriate isotype controls (eBioscience Inc., San Diego CA, USA) by flow cytometry (FACS LSRFortessa; BD Biosciences, San Jose CA, USA). Data were analyzed by BD FACS Diva software.

Proliferation assay. BrdU staining was used to measure cell proliferation by absorbance at 370 nm by using the BrdU Cell Proliferation Assay Kit (Roche, Penzberg, Germany) according to the manufacturer's instruction. In Brief, BMMs from *Sirt6*$^{fl/fl}$ and *Sirt6*$^{fl/fl}$*Mx1Cre* mice were cultured with M-CSF (30 ng/ml) and RANKL(100 ng/ml) for 2 days and BrdU-labeling medium was added to cells and incubated at 37℃ for 2 hours. Cells were washed three times with 1X PBS and anti-BrdU reagent was added for 90 minutes at 37℃. Cells were measured at 370 nm in a microplate reader.

Immunoblot analysis. Cell were washed twice with cold-PBS and lysed in RIPA buffer (10 mM Tris-HCl (pH 8.0), 150 mM NaCl, 1% NP-40, 1 mM EDTA, 0.2% sodium deoxycolate) supplemented with protease inhibitors and phosphatase inhibitor. After incubation for 1 hour on ice, lysates were centrifuged at 14,000 rpm for 20 minutes at 4°C. Subsequently, protein

concentration was measured by Bradford assay (Bio-Rad). Protein samples were resolved by SDS-PAGE and analyzed by immunoblot with antibodies. The following primary antibodies (Cell Signaling Technology) were used: phospho-AKT (9271), phospho-p38 (9219), phospho-JNK (9251), phospho-ERK (9101), AKT (9272), p38 (9212), JNK (9252), and ERK (9102).
